# An engineered tale-transcription factor rescues transcription of factor VII impaired by promoter mutations and enhances its endogenous expression in hepatocytes

**DOI:** 10.1038/srep28304

**Published:** 2016-06-24

**Authors:** Elena Barbon, Silvia Pignani, Alessio Branchini, Francesco Bernardi, Mirko Pinotti, Matteo Bovolenta

**Affiliations:** 1 Department of Life Sciences and Biotechnology, University of Ferrara, Italy; 2 LTTA Center, University of Ferrara, Italy

## Abstract

Tailored approaches to restore defective transcription responsible for severe diseases have been poorly explored. We tested transcription activator-like effectors fused to an activation domain (TALE-TFs) in a coagulation factor VII (FVII) deficiency model. In this model, the deficiency is caused by the −94C > G or −61T > G mutation, which abrogate the binding of Sp1 or HNF-4 transcription factors. Reporter assays in hepatoma HepG2 cells naturally expressing FVII identified a single TALE-TF (TF4) that, by targeting the region between mutations, specifically trans-activated both the variant (>100-fold) and wild-type (20–40-fold) *F7* promoters. Importantly, in the genomic context of transfected HepG2 and transduced primary hepatocytes, TF4 increased *F7* mRNA and protein levels (2- to 3-fold) without detectable off-target effects, even for the homologous *F10* gene. The ectopic *F7* expression in renal HEK293 cells was modestly affected by TF4 or by TALE-TF combinations. These results provide experimental evidence for TALE-TFs as gene-specific tools useful to counteract disease-causing promoter mutations.

Little has been done to counteract the effect of promoter mutations, which represent a relevant cause of human genetic disease (http://www.hgmd.cf.ac.uk), *via* engineered transcription factors (eTFs).

One versatile tool is the transcription activator-like effectors (TALEs) that exhibit simple DNA-binding properties with a conserved central region composed of a series of 33–35 amino acid repetitive elements^1^,^2^, each containing a highly variable di-residue (RVD) at the 12^th^–13^th^ positions that dictates the binding preference^3^,^4^. Therefore, the tailored mutagenesis of the RVDs and fusion to transcriptional activators (i.e., VP16, VP64, p300, p65, TET1)^5^ generates TALE-TFs that can target a selected promoter region and increase gene expression by stimulating transcription^6^ (Fig. 1a). Thus far, TALE-TFs have been mainly exploited to study stem-cell maintenance and differentiation^7^,^8^,^9^,^10^,^11^,^12^,^13^,^14^ or to increase transcription of the frataxin gene in the presence of a trinucleotide (GAA) repeat expansion^15^. Single nucleotide changes severely impairing the transcription of disease genes have not yet been addressed.

Factor VII (FVII) is the serine-protease that triggers blood coagulation, and it offers a unique model of finely regulated gene expression. Therefore, FVII can be taken as model system to study the positive or negative modulation of gene transcription. Here, we chose two mutations known to cause severe FVII deficiency^16^ as paradigmatic examples of mutations that impair transcriptional activity by affecting key transcription factor binding sites, which is a common pathogenic mechanism induced by promoter defects. Through reporter gene assays, we identified a single TALE-TF that was able to rescue the defective *F7* promoter, and it was also active in the context of chromatin in cultured cells and primary hepatocytes. Thus, this work provides evidence of the efficacy of TALE-TFs on rescuing disease-causing promoter mutations.

## Results and Discussion

**Figure 1:**
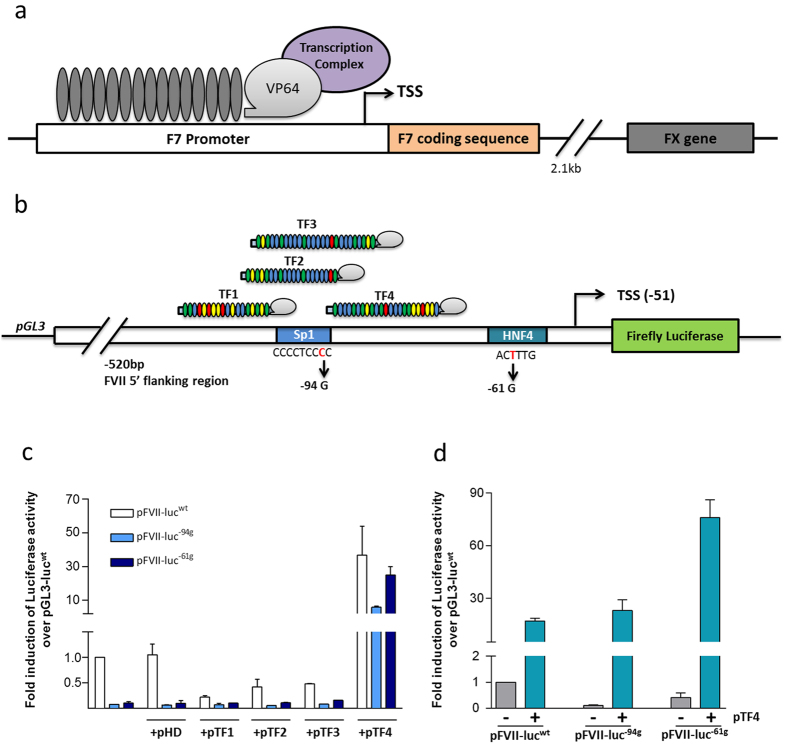
TALE-TF structure and design, schemes of the *F7* gene locus and of the reporter *F7* constructs and transactivation activity of engineered TALE-TFs in reporter gene assays in HepG2 and HuH7 cells. (**a**) Schematic representation of the TALE-TF structure and of the genomic region comprising *F7* and the adjacent *F10* genes. TALE-TF is composed of a TALE-derived modular DNA binding-domain (TALE-DBD) fused with a VP64 trans-activation domain, the latter recruiting the transcriptional machinery (Transcription Complex). (**b**) Representation of the reporter *F7* constructs, which includes the proximal promoter region of *F7* (520 bp) driving the expression of the firefly luciferase. The relative localization of the four TALE-TFs (TF1, TF2, TF3 and TF4) is reported above the scheme. The wild-type and mutated Sp1 and HNF4 binding sequences are indicated below. TSS, Transcription Start Site. (**c**) Promoter activity of *F7* variants alone or upon co-transfection with the pTFs or the pHD, devoid of the TALE-DNA binding domain, in HepG2 cells. (**d**) Promoter activity of *F7* variants alone or upon co-transfection with the pTF4 in HuH7 cells. Histograms report the fold expression of normalized luciferase activity over that of the pFVII-luc^wt^. The results are expressed as the mean ± standard deviation from at least three independent experiments.

The −94C > G and the −61T > G homozygous promoter mutations are known to cause severe FVII deficiency by impairing the binding of the Sp1 and HNF-4 transcription factors^17^,^18^, respectively (Fig. 1b). Using computational analyses^19^, we designed four TALE-TFs (TF1-4) targeting sequences 18–26 bp in length within the 70 bp region between positions −122 to −68 of the *F7* proximal promoter (Fig. 1b), which are predicted to be unique in the human genome (Table S1). The TALE-derived DNA binding domains were expressed as a fusion protein with the VP64 effector domain (Fig. 1a). VP64 is a powerful and versatile trans-activator because its activity can be modulated by altering the number of VP domains^5^,^8^,^9^,^13^.

Screening conducted using reporter gene assays in FVII-expressing hepatoma cells (HepG2) identified a single TALE-TF (TF4), which was designed to target the *F7* promoter sequence ^5′^TCCCTCTGTCACCCTTGGAGGC^3′^ between the Sp1 and HNF-4 binding sites, that showed trans-activation features. Specifically, TF4 remarkably increased the transcriptional activity of the severely impaired *F7* promoter variants to an extent significantly higher than that of the wild-type construct (pFVII-luc^−94g^, 5.9 ± 0.6-fold; pFVII-luc^−61g^, 25 ± 4.9-fold). The pFVII-luc^wt^ luciferase activity was also significantly enhanced (pFVII-luc^wt^, 36.6 ± 17.3-fold) (Fig. 1c). Taking into account that tumour cells show different transcriptional mechanisms and profiles^20^, TF4 activity was also explored in HuH7 hepatoma cells. In this context, TF4 exhibited robust trans-activation activity on *F7* promoter variants (pFVII-luc^−94g^, 23 ± 6.1-fold; pFVII-luc^−61g^, 76 ± 10.2-fold) and wild-type (17.1 ± 1.5-fold) (Fig. 1d). Conversely, TF1, TF2 and TF3, which were designed to target upstream regions (Fig. 1b), were ineffective in rescuing *F7* promoter variants and seemed to exert an inhibitory effect (Fig. 1c). These results may be due to an underlying competition and/or steric hindrance with the endogenous transcription factors on their binding sites.

**Figure 2:**
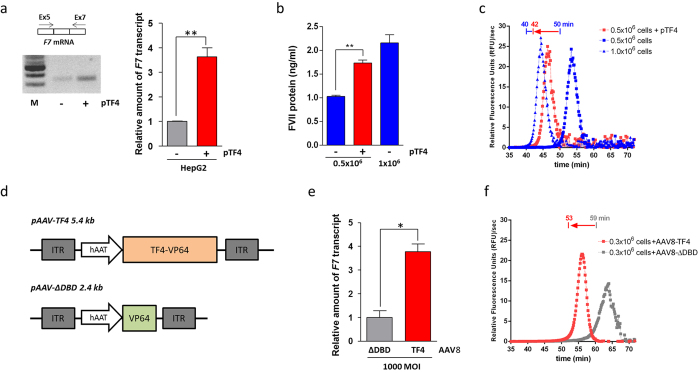
TF4 trans-activation activity on the *F7* gene in human hepatoma HepG2 cells and in human hepatocyte Hep10 cells. (**a**) Results from conventional RT-PCR analysis of *F7* mRNA isolated from HepG2 cells (left side) transfected with pTF4 (+) or untransfected cells (−). The amplicons were separated on a 2% agarose gel. M, molecular weight marker. The right panel reports the RT followed by the qPCR analysis of *F7* mRNA in HepG2. (**b**) Histograms reporting the FVII protein levels in medium from 0.5 × 10^5^ HepG2 cells transiently transfected with TF4 (1.74 ± 0.06 ng/ml, red bar), which were compared with those of the medium from 0.5 × 10^5^ and 1.0 × 10^5^ (blue bars) untransfected HepG2 cells basally expressing 1.03 ± 0.06 and 2.16 ± 0.17 ng/ml FVII, respectively. (**c**) FXa generation activity in FVII-deficient plasma supplemented with medium from HepG2 cells. The graph reports the first derivative of relative fluorescence units (RFU) as a function of time, and the lag times (indicated above the curves) were extrapolated from 0.5 × 10^5^ cells transfected with pTF4 (42 minutes, red square) or untransfected cells (50 minutes, blue square). The lag time relative to 1.0 × 10^5^ HepG2 control cells is also reported (40 minutes, blue triangle). (**d**) Scheme representing the AAV8-based vectors expressing the TF4 (pAAV-TF4) or a variant lacking the DNA binding domain (pAAV-ΔDBD) under the control of the liver specific human alpha-1 antitrypsin (hAAT) promoter. (**e**) Histogram reporting the qPCR analysis of *F7* mRNA in Hep10 cells transduced with AAV8-TF4 and –ΔDBD at 1000 MOI. (**f**) FXa generation activity in FVII-deficient plasma supplemented with medium from transduced Hep10 cells. The red arrow indicates the shortening of lag times induced by treatment with AAV8-TF4, compared to the AAV8-ΔDBD. The data are expressed as the mean ± standard deviation, and analysed by Student’s t-test (*p < 0.05; **p < 0.01).

As hepatocytes from the severely affected FVII-deficient patients with promoter mutations were unavailable, we tested TF4 on the endogenous *F7* gene expression in HepG2 cells. RT-PCR analysis conducted on the total RNA extracted from pTF4-transfected cells revealed an increased amount of *F7* mRNA compared to control cells, which corresponded to a 3.6 ± 0.3-fold increase upon qPCR (Fig. 2a). Intrigued by this observation, we investigated the impact of TF4 on FVII protein expression. For untransfected HepG2 cells, FVII expression is directly related to the cell number (~1 and 2 ng/ml from 0.5 × 10^6^ and 1.0 × 10^6^ cells, respectively) (Fig. 2b, blue bars). In medium with 0.5 × 10^6^ cells transfected with pTF4, we observed approximatively a two-fold increase of FVII antigen levels (Fig. 2b, red bar), which was consistent with the extent of the increase in the *F7* mRNA levels. Most importantly, the expressed FVII was enzymatically active, as measured by a very sensitive functional fluorogenic assay in plasma that evaluates the activation of factor X, the natural substrate of FVII^21^. In this assay, the activity of FVII responsible for triggering the coagulation cascade process inversely correlates with lag time^22^. As shown in Fig. 2c, in medium with 0.5 × 10^6^ cells transfected with pTF4, we detected an increase of secreted protein levels with a concurrent shortening of lag times relative to untransfected cells (42 vs 50 minutes). These results were comparable to those from 1.0 × 10^6^ control cells (Fig. 2c).

This coherent increase in *F7* mRNA, secreted protein and activity levels observed upon pTF4 treatment appears to be remarkable in the light of the very poor transfection efficiency (approximately 5%) of HepG2 cells, as estimated by FACS analysis (GFP tracer encoded by the pTF4 vector; data not shown).

Certainly, the efficacy of TALE-TFs strongly depends on the chromatin status and the accessibility of the target regions^23^, and this issue is one that cannot be properly assessed in hepatoma cells and through reporter gene assays exploiting episomal vectors. Thus, we transduced Hep10 human primary hepatocytes using adeno-associated viral vectors expressing the entire TF4 (AAV8-TF4) or a variant lacking the DNA binding domain (AAV8-ΔDBD) as a control (Fig. 2d).

The RNA analysis showed a 3.7 ± 0.33-fold induction of *F7* gene expression in AAV8-TF4 versus AAV8-ΔDBD transduced Hep10 cells (Fig. 2e). FXa generation assays in medium revealed a significant shortening of lag times between AAV8-TF4 and AAV8-ΔDBD transduced cells, indicating an increase in the levels of secreted FVII protein able to promote the generation of FXa in plasma systems (Fig. 2f).

Similar to HepG2 cells, the transactivation of the endogenous *F7* transcription in Hep10 cells may be limited by competition with the HNF4 transcription factor. Additionally, transactivation could be further limited by the rate of both infection and transgene expression of AAV vectors *in vitro*^24^,^25^. However, from a translational therapeutic perspective, the use of AAV is very promising^26^,^27^,^28^.

**Figure 3:**
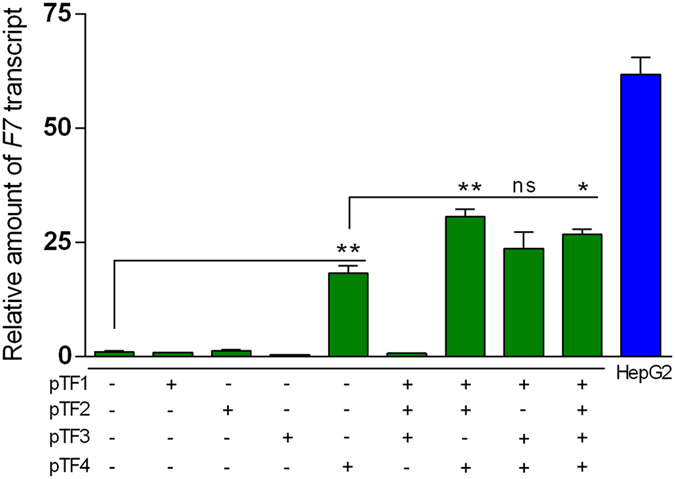
Trans-activation activity of TF1-4, individually or in combination, on *F7* gene in renal HEK293 cells. The histograms report the relative amount of F7 mRNA detected by qPCR analysis in HEK293 cells transfected with a single TALE-TF or combinations of TALE-TFs compared to endogenous F7 expression in HepG2 cells. The data analyses were performed with Student’s t-test comparing pTF4-treated cells with untreated cells (**p < 0.01) and the combined TFs with the pTF4-treated cells (*p < 0.05; **p < 0.01, ns, not significant).

To investigate differences in the target region accessibility caused by different chromatin statuses, the effect of TFs was also explored in human embryonic kidney 293 cells (HEK293). Because HEK293 cells are of kidney origin, they lack the liver-specific HNF4 transcription factor and do not significantly express FVII. This system enabled us to provide insights into both the TF4 mechanism of transcription activation and the steric hindrance hypothesis in HepG2 cells for the TFs 1–3. Even in this experimental setting, only TF4 was able to significantly increase the transcriptional levels of the endogenous *F7* gene (Fig. 3). These findings point to a mechanism in which TF4 activity is not mediated by HNF-4, in accordance with the known ability of VP64 to recruit the basal transcriptional machinery^1^,^29^.

Moreover, these data support the hypothesis that TF4 activity depends on its position and the accessibility to the target region. In fact, in the absence of the HNF-4 transcription factor, the single TFs 1–3, which previously inhibited the activity of the FVII-luc^wt^ construct potentially through competition with endogenous transcription factors, were unable to trans-activate *F7* gene expression in HEK293 cells (Fig. 3). Noticeably, by transfecting pTF4 in combination with the other TALE-TFs, we detected an additionally increased expression of the endogenous *F7* (>20-fold), which was modest compared to that in untreated HepG2 cells. Consistently, the combination of TF1-3 was ineffective (Fig. 3).

Taken together, the observed synergistic effects note that TF1-3 could bind to their target, but they may bind too far from the transcription start site to enhance gene expression.

**Figure 4:**
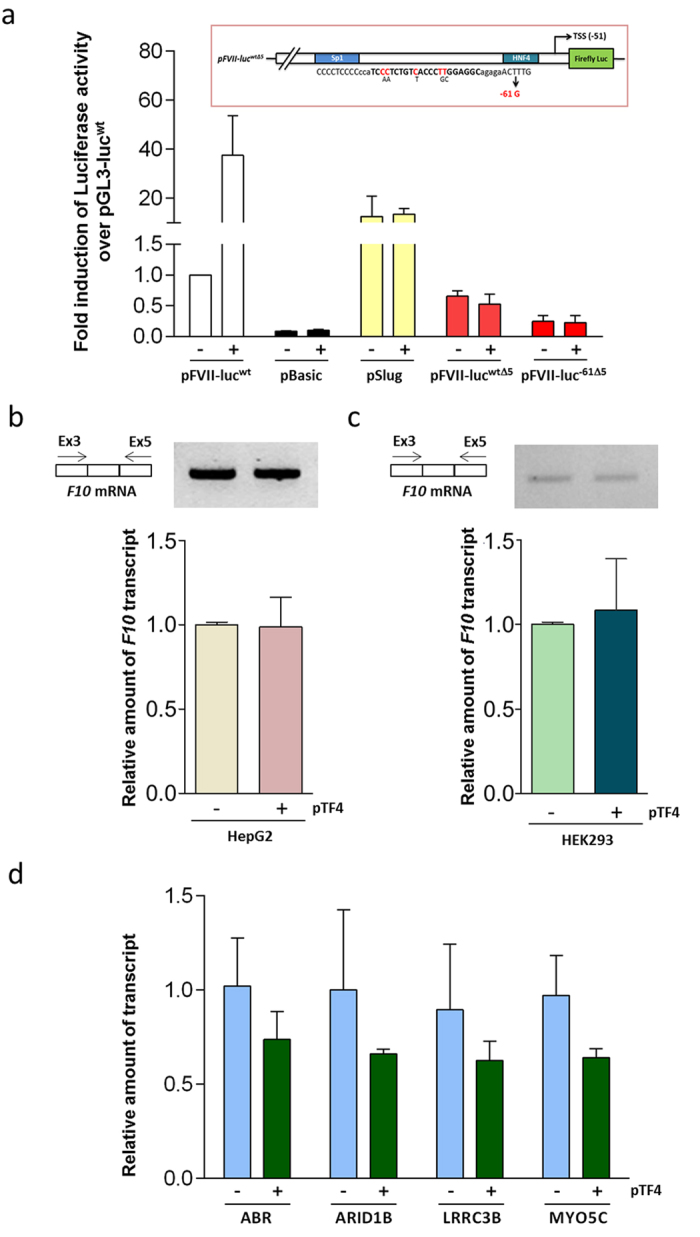
Evaluation of TF4 specificity and off-targets. (**a**) Trans-activation effects of pTF4 on the expression of pBasic (lacking the promoter) pSlug (carrying the unrelated ubiquitous Slug promoter) or *F7* promoter variants with the mutated (5 out of 22 bp) TF4 target sequence (pFVII-luc^wtΔ5^, pFVII-luc^−61Δ5^) in HepG2 cells. (**b,c**) Results from conventional RT-PCR and RT followed by qPCR analysis of *F10* mRNA isolated from HepG2 (**b**) or HEK293 (**c**) cells transfected with TF4 (+) or untransfected cells (−). The amplicons were separated on 2% agarose gel. (**d**) Relative transcript quantification of the predicted off-targets genes by qPCR. The data analyses were performed with Student’s t-test comparing TF4-treated cells with untreated cells. The results are expressed as the mean ± standard deviation from at least three independent experiments.

Target specificity is a crucial issue when proposing a new therapeutic approach. Thus, we assessed our designed TALE-TFs in multiple control experiments for off-target effects. The key role of the TALE-DNA binding domain was to guarantee specific targeting. This specificity was demonstrated in control experiments where the VP64 activation domain was expressed alone. Negligible effects on the expression of the pHD plasmid were observed in these experiments (Fig. 1c). In complementary experiments, TF4 was unable to increase the transcription of neither the pBasic construct, which does not have a promoter, or the pSlug vector, which possesses a ubiquitous and *F7*-unrelated Slug promoter^30^ (Fig. 4a). Most importantly, the TF4 efficacy was abolished upon the partial mutagenesis (five out of the 22 nucleotides) of the target sequence (Fig. 4a, inset), either in the wild-type (pFVII-luc^wtΔ5^) or mutated (pFVII-luc^−61Δ5^) contexts, a finding that supports high specificity and a reduced probability of off-target effects (Fig. 4a).

The *F7* model also offered us the opportunity to test, in the context of chromatin, the specificity of TF4 for activation of *F7* over the neighbouring and homologous *F10* gene, which is located approximately 2.1 kb downstream of the *F7* locus (Fig. 1a) and exhibits a 15-fold higher expression level than FVII. Our results with RT-PCR and qPCR analyses indicated that TF4 exerted no effect on *F10* transcription in both HepG2 (Fig. 4b) and in HEK293 cell lines (Fig. 4c).

To investigate in detail the possibility of TF4 off-targeting, we tested the expression of the four genes showing the highest score of off-target prediction (supplementary Table S1). qPCR performed in pTF4-treated versus untreated HepG2 indicated no significant influence on these targets (Fig. 4d), which rules out unspecific effects on the most probable candidates.

Taken together, these results obtained with complementary approaches demonstrate the ability of TF4 to specifically enhance the *F7* gene promoter function, which leads to a concurrent increase of secreted FVII protein and activity levels. Taking into account the very low therapeutic threshold for FVII deficiency^31^, this enhancement could rescue coagulation and reduce haemorrhagic diathesis in severe patients. It is worth noting that none of the promoter mutations identified so far in FVII deficient patients (http://www.hgmd.cf.ac.uk) fall within the region targeted by the active TF4. This observation implies that TF4 is applicable for all *F7* promoter mutations; thus, cohorts of patients could benefit without any modification of the TALE DNA binding domain.

We believe that these novel data lay a robust foundation for the further manipulation of the TALE-TF construct to control the strength or mechanism of the activation domain (i.e., number of VP repeats^13^,^32^ or different domains^5^) as well as its expression levels (promoter strength) and tissue specificity (i.e., liver specific promoters^33^). Although we provided evidence for the successful use of TF4 delivered by an AAV vector with liver tropism only in a cellular model^34^, this result could lead to optimized TF4-derivatives that could be used to increase specificity and prevent possible FVII over-expression and consequent thrombotic risks.

In conclusion, to the best of our knowledge, this study provides the first experimental insights into the ability of a single engineered TALE-TF to rescue, with negligible off-target effects, gene transcription impaired by two promoter mutations. If delivered *in vivo*, these results could open new therapeutic strategies for coagulation factors as well as other genetic diseases sharing similar pathogenic mechanisms.

## Methods

### Design and cloning of the expression vectors

To create the pFVII-luc^wt^ vector, the *F7* proximal promoter region (520 bp) was amplified with primers ^5′^AAAAAGCTTCTGTGGCTCACCTAAGAAACCAG^3′^ and ^5′^AAAAAGCTTGATGAA ATCTCTGCAGTGCTGC^3′^ and cloned upstream of the firefly luciferase sequence into the pGL3 Basic Vector (Promega, Madison, USA) using the Hind III restriction sites (underlined).

The pFVII-luc variants were created by mutagenesis (QuickChange II Site-Directed Mutagenesis Kit, Agilent Technologies, Santa Clara, USA) of pFVII-luc^wt^ using forward primers ^5′^TCCTCCCCTCCGCCATCCCTCTG^3^ (pFVII-luc^−94g^), ^5′^GCAGAGAACGTTGCCCGTCAGTC^3′^ (pFVII-luc^−61g^) and ^5′^CCCCCATCCCTCTGTTGGAGGCAGAGAA^3′^ (pFVII-luc^wtΔ5^ and pFVII-luc^−61Δ5^). The reverse primers were complementary to the forward primers.

TALE-TFs (named pTF1-4) were designed using the TAL Effector Targeter webtool^19^ entering the 150 bp of the minimal promoter region of the F7 gene, scanning for repeat arrays of 18–26 TALE and T as an upstream base and unchecking all the other options.

Four TALEs binding to the minimal promoter sequence and selected upon the minimal number of off-targets were created as described^32^.

### Cell cultures and reporter gene assays

The FVII studied in this work is of liver origin, so the reporter gene assays were conducted in hepatoma HepG2 and HuH7 cells. Cells were seeded into 12-well plates (0.2 × 10^6^ cells/well) and transfected using Lipofectamine 3000 (Life Technologies, Carlsbad, USA) with 1 μg of each pFVII-luc variant and/or 1 μg of each pTF or pHD, expressing the VP64 alone, as a control. To normalize for the transfection efficiency, the cells were co-transfected with 100 ng of pRluc (Promega) expressing Renilla luciferase. Forty-eight hours post-transfection, cells were lysed to evaluate the luciferase activity by the Dual-Luciferase assay (Promega).

To evaluate transfection efficiency, FACS analysis was conducted in HepG2 cells transfected with TALE-TFs vectors, which express GFP fused to the 3^′^ of the TALE-TF genes by a self-cleaving P2A linker.

### AAV Plasmid construction and packaging

TF4 or the ΔDBD (TF without the DNA binding domains) backbones were cloned in the AAV vector plasmid under the liver specific chimeric promoter of human alpha-1-antitrypsin and cer/hepatic locus control region^25^ and packaged by ViGene Biosciences, Inc., as AAV8 strain. The titers reuslted 3.61 × 10^14^ GC/ml for the AAV8-TF4 and 3.15 × 61 × 10^14^ GC/ml for the AAV8-ΔDBD.

### Primary hepatocyte transduction

Hep10 cells (human hepatocytes from ten donors) were obtained from Thermo Fisher Scientific (Waltham, USA) and were cultured according to the manufacturer’s instructions. Briefly, one vial of Hep10 cells was thawed, diluted in Cryopreserved Hepatocytes Recovery Medium (CHRM), centrifuged at 100 rcf for 10 minutes at 4 °C and resuspended in Williams’ Medium E (1x, no phenol red) supplemented with the Hepatocyte Maintenance Supplement Pack. Twenty-four hours later, cells were counted and seeded in 12-well plates at a density of 0.3 × 10^6^ cells/ml in Williams’ Medium E and the Supplement pack as described above, with the addition of Vitamin K 5 μg/ml and the AAV8-TF4 or pAAV8-ΔDBD at 1000 MOI. Forty-height hours later, the cells and medium were collected for the analysis of RNA and FVII activity levels.

### RNA extraction and cDNA synthesis

HepG2, Huh7, HEK293 and Hep10 cells were transfected as described above. Forty-eight hours later, the cells were collected and the total RNA was isolated using TRIzol reagent (Life Technologies) to generate cDNA through the iScript cDNA Synthesis Kit (Bio-Rad Laboratories, Hercules, USA).

### mRNA analysis of F7 and F10 genes

*F7* and *F10* transcripts were detected by RT-PCR and quantitative PCR (qPCR), with primers Ex5F ^5′^GAGAACGGCGGCTGTGAG^3′^, Ex7R ^5′^GTTCCTCCAGTTCTTGATTTTGTCG^3′^ and primers Ex3F ^5′^AATGAATTCTGGAATAAATACA^3′^, and Ex5R ^5′^CTGTGGGAATGCAGGCCTTGC^3′^, respectively. RT-PCR was performed with AmpliTaq Gold 360 (Thermo Fisher Scientific) for 35 cycles, whereas qPCR employed the SsoFast EvaGreen Supermix (Bio-Rad) for 40 cycles. GAPDH and 18S were considered as housekeeping genes. The calculation of the fold changes was based on the 2^−ΔΔCT^ method (Applied Biosystems User Bulletin #2).

### Evaluation of FVII protein levels and activity

The protein levels for FVII were evaluated by ELISA (Cedarlane, Burlington, Ontario, Canada) in medium collected 48 hours post-transfection. Known concentrations of plasma-derived FVII (Haematologic Technologies Inc., Essex Junction, VT, USA) were used to create the reference curve.

Activated factor X (FXa) generation assays were performed as described^21^. Medium from transfected cells was added 1:1 (vol/vol) to commercial FVII-deficient plasma (BioMedical Inc., George King, USA), adjusted to a final dilution of 1:20 in a dilution buffer (20 mM HEPES, 150 mM NaCl, 0.1% PEG-8000, pH 7.4) and sub-sampled in a 96-well microplate (Costar, Corning, NY, USA). Coagulation was triggered by the addition of a mixture containing a specific fluorogenic substrate for FXa (150 μM, American Diagnostica Inc., Greenwich, USA) and 1/100 Innovin (Siemens Healthcare, Marburg, Germany) prepared in a reaction buffer (20 mM HEPES, 150 mM NaCl, 5 mM CaCl_2_, 0.1% PEG-8000, pH 7.4). The generation of FXa was measured as fluorescence emission (Relative Fluorescence Units, RFU; 360 nm excitation, 465 nm emission) over time on a microplate fluorimeter (Fluoroskan Ascent FL, Thermo Fisher Scientific, Helsinki, Finland).

FXa generation assays were analysed by the statistical software GraphPad Prism 5 (GraphPad Software, San Diego, USA). The specific parameter lag time (expressed in minutes) in FXa generation assays was extrapolated from the first derivative of relative fluorescence units (RFU) as a function of time (minutes).

### Off-target analysis

TALE-TFs off-target analysis was performed by entering the target region for each TALE-TF in the Target Finder software^19^ with default options. The first ten off-target regions for each TF are reported with the prediction score in the supplementary Table S1. The off-target sites for the TF4 were analysed by qPCR as described above, with the following primers:

ABR_F, CTGCTGAGGAAACACCACAG; ABR_R, CCCCACAACTTGCTCCTAAG; LRRC3B_F, GCCCAAGCAAGGAAAGAAAT; LRRC3B_R, GGTAAAGGTTCCAGCCCTGT; ARID1B_F, GCGTGTGCAGGAGTTCAATA; ARID1B_R, GGAATTTCCATCTTGCTCTCA; and MYO5C_F, TCCTAAATAGCCGGGAGGAT; MYO5C_R, CTGCTGGGGATCTGAATCAT.

## Additional Information

**How to cite this article**: Barbon, E. *et al*. An engineered tale-transcription factor rescues transcription of factor VII impaired by promoter mutations and enhances its endogenous expression in hepatocytes. *Sci. Rep.* **6**, 28304; doi: 10.1038/srep28304 (2016).

## Supplementary Material

Supplementary Information
