# Supplementary material for: An engineered tale-transcription factor rescues transcription of factor VII impaired by promoter mutations and enhances its endogenous expression in hepatocytes
Source: Sci Rep. 2016 Jun 24;6:28304. doi: 10.1038/srep28304 (PMC4920032; doi:10.1038/srep28304)
Supplement: Supplementary Information [file srep28304-s1.pdf]

AN ENGINEERED TALE-TRANSCRIPTION FACTOR RESCUES TRANSCRIPTION OF FACTOR VII IMPAIRED BY PROMOTER MUTATIONS AND ENHANCES ITS ENDOGENOUS EXPRESSION IN HEPATOCYTES

Elena Barbon[1], Silvia Pignani[1], Alessio Branchini[1,2], Francesco Bernardi[1,2], Mirko Pinotti*[1,2] and Matteo Bovolenta*[1]

[1] Department of Life Sciences and Biotechnology, University of Ferrara, Italy.

[2] LTTA Center, University of Ferrara, Italy.

***Corresponding authors**

Correspondence to:

-Matteo Bovolenta, Department of Life Sciences and Biotechnology, University of Ferrara, Via Fossato di Mortara 74, 44121, Ferrara, Italy.

Phone +39 0532 974485 Fax +39 0532 974485 E-mail: bvlmtt@unife.it

-Mirko Pinotti, Department of Life Sciences and Biotechnology and LTTA, University of Ferrara, Via Fossato di Mortara 74, 44121, Ferrara, Italy.

Phone +39 0532 974424 Fax +39 0532 974424 E-mail: pnm@unife.it

Short title: Rescue of defective FVII promoter by TALE-TFs

TALE	Chromosome	Strand	Score	Start Position	Position	Target Sequence
TF1	13	Plus	6.41	113760050	82 bp upstream F7 TSS	T GTCCTCCCCTCCCCCATCCCTCTGT
	3	Minus	14.03	131763609	CPNE4 gene intron 2	T CTCCTCCCCTCCACCCTCCCTCCAT
	7	Minus	14.99	33896418	Intergenic	T CTCCTCCCCGCCCCAACCCCTCTAT
	17	Plus	15.21	64972423	AMZ2P1 gene intron 1	T GTCCTCCCCTCCCTCCTCCCTCTGT
	21	Minus	15.52	43210963	Intergenic	T TCCCTCCCCTCCCCCATCCCTCCCC
	5	Plus	15.80	38806449	OSMR-AS1 intron 1	T CTCCTCCCCACCCCCAAACCCCTGT
	2	Plus	15.81	218935593	Intergenic	T CTCCTCCCCTTCCCCATCCCTCTCC
	4	Minus	15.84	73893665	Intergenic	T CTCCTCCCCTCCCCCCACCCTATGA
	9	Minus	15.97	6016354	Intergenic	T ATCCTCCCCTCCCCCCAACCTCTGG
	2	Minus	16.08	224591346	Intergenic	T CCCCTCCCCTCCCCCCTCCCTCTCT
	6	Minus	16.08	30930728	Intergenic	T CCCCTCCCCTCCCCCCTCCCTCTCT
TF2	13	Plus	4.91	113760048	90 bp upstream F7 TSS	T GTGTCCTCCCCTCCCCCAT
	1	Plus	6.17	119729206	PHGDH gene intron 4	T CTATCCTCCCCTCCCCCAT
	22	Plus	6.91	35345891	TOM1 gene intron 13	T GTGTCCTCCCCTCCCCCAA
	9	Minus	6.91	98066084	NANS gene intron 2	T GTGTCCACCCCTCCCCCAT
	20	Plus	7.03	60006851	CDH26 gene intron 3	T CTCTCCTCCCCTCCCCCAT
	12	Minus	7.11	53892527	Intergenic	T GCATCCTCCCCTCCCCCAT
	16	Minus	7.11	2821113	PRSS21 gene intron 5	T GTATCCTCCCCTCCCCCAA
	5	Plus	7.11	138522044	ETF1 gene intron 2	T ATGTCCTCCCCTCCCCCAC
	4	Plus	7.15	170949261	Intergenic	T ATTTCCTCCCCTCCCCCAT
	7	Minus	7.24	41713137	INHBA-AS1 intron 2	T GTGTCCTCCCCTCCCACAT
	12	Plus	7.97	5698467	ANO2 gene intron 14	T GCCTCCTCCCCTCCCCCAT
TF3	13	Plus	7.27	113760067	105 bp upstream F7 TSS	T TCAGAGGACGCCTGTGT
	11	Plus	8.03	105792362	GRIA4 gene intron 4	T TCAGAAAACACCTGTAT
	20	Plus	8.03	22362793	Intergenic	T TCAAAGAACACCTGTAT
	4	Minus	8.03	60013525	Intergenic	T TCAAAAGACACCTATGT
	3	Plus	8.23	88515341	Intergenic	T TCAGAAAACACCTATAT
	11	Plus	8.43	100721297	ARGHAP42 gene intron 1	T TCAAAAAACACCTATAT
	1	Minus	8.49	5781418	Intergenic	T TCAGAGAACCCCTGTGT
	1	Plus	8.69	119102891	Intergenic	T TCAGAGGACACCTATCT
	10	Plus	8.69	59870048	CCDC6 gene intron 1	T TCAGAAGACCCCTATGT
	4	Plus	8.69	189555373	Intergenic	T TCAGAACACGCCTATGT
	9	Plus	8.69	91979099	Intergenic	T TCAAAGCACACCTGTGT

	13	Plus	7.27	113760067	69 bp upstream F7 TSS	T CCCTCTGTCACCCTTGGAGGC
	20	Plus	9.71	61061976	Intergenic	T CCCTCTTTCACCCTTAAAGGC
	6	Plus	10.72	157285170	ARID1B gene intron 5	T CTCTCTGTCACCCTTGGAAGC
	17	Plus	10.81	1050309	ABR gene intron 1	T CCCTCTGTGACCCTTGGAGGC
	14	Plus	10.93	44336196	Intergenic	T CCCTCTGTCACCCTAAAAACC
TF4	3	Plus	11.79	26688704	LRRC3B gene intron 1	T CCCTCTGTCACCCTCCAACAC
	9	Minus	11.84	91324087	Intragenic	T CCCTCTCTCACCCTTGGGGGC
	5	Plus	11.88	174305929	Intergenic	T CCCTCTACCACCCTAGAAGAC
	8	Plus	11.92	47027323	Intergenic	T CCCTCTACCACCCTTAAAGTC
	15	Plus	12.34	52533969	MYO5C gene intron 20	T CCCTCTGTCACCCACGCAGGC
	1	Plus	12.38	71040326	Intergenic	T CCCTCTGTCACCCTGCCAGGC

Supplementary Table S1: On-target and off-target chromosomal coordinates and scores for each TALE-TF predicted with the online tool TAL Effector Targeter[19].
